# Human Cryptochrome-1 Confers Light Independent Biological Activity in Transgenic *Drosophila* Correlated with Flavin Radical Stability

**DOI:** 10.1371/journal.pone.0031867

**Published:** 2012-03-12

**Authors:** Jacqueline Vieira, Alex R. Jones, Antoine Danon, Michiyo Sakuma, Nathalie Hoang, David Robles, Shirley Tait, Derren J. Heyes, Marie Picot, Taishi Yoshii, Charlotte Helfrich-Förster, Guillaume Soubigou, Jean-Yves Coppee, André Klarsfeld, Francois Rouyer, Nigel S. Scrutton, Margaret Ahmad

**Affiliations:** 1 Université Paris VI, Paris, France; 2 Manchester Interdisciplinary Biocentre and Faculty of Life Sciences, University of Manchester, Manchester, United Kingdom; 3 Institut de Neurobiologie Alfred Fessard, CNRS UPR 2216 (NGI), Gif-sur-Yvette, France; 4 Graduate School of Natural Science and Technology, Okayama University, Okayama, Japan; 5 University of Würzburg, Biocentre, Neurobiology and Genetics, Würzburg, Germany; 6 Institut Pasteur, Transcriptome and Epigenome Platform, Genomes and Genetics Department, Paris, France; 7 Penn State University, Media, Pennsylvania, United States of America; Cinvestav, Mexico

## Abstract

Cryptochromes are conserved flavoprotein receptors found throughout the biological kingdom with diversified roles in plant development and entrainment of the circadian clock in animals. Light perception is proposed to occur through flavin radical formation that correlates with biological activity *in vivo* in both plants and *Drosophila*. By contrast, mammalian (Type II) cryptochromes regulate the circadian clock independently of light, raising the fundamental question of whether mammalian cryptochromes have evolved entirely distinct signaling mechanisms. Here we show by developmental and transcriptome analysis that *Homo sapiens* cryptochrome - 1 (HsCRY1) confers biological activity in transgenic expressing *Drosophila* in darkness, that can in some cases be further stimulated by light. In contrast to all other cryptochromes, purified recombinant HsCRY1 protein was stably isolated in the anionic radical flavin state, containing only a small proportion of oxidized flavin which could be reduced by illumination. We conclude that animal Type I and Type II cryptochromes may both have signaling mechanisms involving formation of a flavin radical signaling state, and that light independent activity of Type II cryptochromes is a consequence of dark accumulation of this redox form *in vivo* rather than of a fundamental difference in signaling mechanism.

## Introduction

Cryptochromes are flavoprotein receptors characterized by striking sequence and structural homology to photolyases, a class of light sensing DNA repair enzyme. Cryptochromes retain the light sensing N-terminal domain of photolyases, but have largely lost DNA repair activity and instead developed novel roles in signaling [1], [2]. Like photolyases, cryptochromes can bind both folate and flavin chromophores and undergo photochemical modification in response to light. In animals, there are two principal families of cryptochromes that have known roles in signaling, namely the Type I and the Type II cryptochromes, occurring in both vertebrates and invertebrates. These have significant homology to the 6-4 photoproduct repair class of photolyases and have roles in both the invertebrate and vertebrate circadian clock [3], [4]. The N-terminal (photolyase-like) domains are highly conserved across both Type I and Type II cryptochromes, and the photolyases, and are thought to be involved in substrate recognition as well as light sensing, while the C-terminal region mainly modulates accessibility of the N-terminal domain to partner molecules [5], [6]. Although much is known of the signaling pathways and interaction partners of both Type I and Type II cryptochromes and of their role in the different animal circadian clocks, the question of the light responsivity of animal cryptochromes, and the mechanism of converting the light signal into a biological response remains to be resolved. This is particularly the case for the Type II cryptochromes found in mammalian systems, including man [7].

It is currently known that animal type I cryptochromes, as exemplified by *Drosophila* cryptochrome (DmCRY), show robust light-dependent responses both in whole living flies and in cell culture systems [1]. DmCRY responses are observed in the blue/near-UV region of the visible spectrum (below 500 nm) with sharp cut-off above 500 nm and a peak at 450 nm, which corresponds closely with the absorption spectrum of oxidized flavin in the visible spectrum [8], [9]. Purified isolated Dmcry proteins are bound to oxidized flavin and can be reduced by light to the anionic radical flavin form [10], [11], which correlates with biological activity [9]. This leads to the suggestion that light activation of animal Type I cryptochromes may occur by a mechanism similar to plant cryptochromes, in that the receptor cycles between inactive (oxidized flavin) and active (radical flavin) states that differentially interact with signaling partners [12], [13]. Further evidence for such a mechanism is obtained from fluorescence and EPR spectroscopic studies of whole cell cultures overexpressing Dmcry, which indicate oxidized to radical flavin interconversion subsequent to illumination [9]. The biological role of DmCRY is therefore assigned primarily as a light sensor to the circadian clock. Nevertheless, some isolated reports of possible dark function of DmCRY, (e.g. during functioning of the antenna clock or of roles in temperature compensation), raise the question of whether there can be additional roles of Type I cryptochrome that do not require illumination [14], [15] and may act by unrelated molecular mechanisms [15], [16].

In marked contrast, Type II cryptochromes are central components of the mammalian circadian oscillator where their role as transcriptional repressor occurs entirely independently of light. This is shown by studies with cryptochrome knockout mice, which demonstrate that in whole organisms, cryptochromes are required for rhythmicity of the circadian clock in constant darkness [17]–[20]. The same is true of rhythmicity in mammalian cell cultures [21], where cryptochrome is required for clock function resulting from non-photic entrainment stimuli such as serum shock [22], [23]. Type II cryptochromes from a variety of organisms, including insects, function as transcriptional repressors in transfection assays, suggesting a marked degree of conservation in function across species lines [24]. However, unlike the function of type I cryptochromes in such assays, transcriptional activity by Type II cryptochromes occurs entirely independently of light. Type II cryptochromes are also not subject to proteolysis subsequent to illumination in S2 insect cell expression assays as are Type I cryptochromes [8], [24]. raising the question whether this class of ‘blue light receptor’ is indeed capable of responding to light at all.

Recently however, a number of studies have shown the possibility of light responsivity also in Type II cryptochromes. Firstly, studies of whole cell cultures overexpressing human (HsCRY1) cryptochrome have shown by both fluorescence and EPR spectroscopies that Type II cryptochromes can indeed undergo photoreduction from oxidized to radical flavin states *in vivo*. Furthermore, illumination of Type II cryptochrome in recombinant flies leads to rapid degradation, indicating a light-induced lability similar to that of Type I cryptochrome (DmCRY) in this same system [9]. Finally, recent reports have shown that Type II cryptochrome from the Monarch Butterfly and human (HsCRY1) can rescue light-dependent magnetoreception in transgenic *Drosophila*, supporting the notion that light sensing can occur in Type II cryptochromes [25], [26].

In this work, the question of the distinction between Type I and Type II cryptochromes is re-examined using a two-fold approach. Firstly, the function of *Homo sapiens* cryptochrome (HsCRY1) was analyzed in *Drosophila*, a small organism that is transparent to light and therefore more amenable to photobiological approaches than cryptochrome-deficient mice, which are for the most part impermeable to light (they are dense animals and covered in hair) and behaviourally and developmentally far more complex. Since cryptochromes are highly conserved across species lines (as is their function as transcriptional repressor of certain promoters [1], it was reasoned that homologs of appropriate signaling intermediates are likely to be recognized and targeted by HsCRY1 even in a heterologous system such as *Drosophila*. The goal was to determine the light dependence of biological phenotypes conferred by this transgene, thus determining whether HsCRY1 could function primarily as a light sensor in an appropriate context. As a corollary to these studies of biological activity in transgenic flies, HsCRY1 protein was purified and evaluated *in vitro* for ability to undergo photochemical modifications in response to light. The goal was to evaluate whether existing models of cry activation through formation of a flavin radical signaling state could be reconciled with HsCRY1 photochemistry. As a result of these two complementary and distinct approaches, the molecular basis for the different light sensitivities of Type I and Type II cryptochromes was correlated with the stability of flavin radical in the purified proteins, consistent with a fundamentally similar mechanism of light activation. As an unexpected additional finding, novel functions for both TypeI and TypeII cryptochromes have been discovered which are unrelated to previously known roles in the circadian clock.

## Results

### Expression of HsCRY1 in transgenic flies

**Figure 1:**
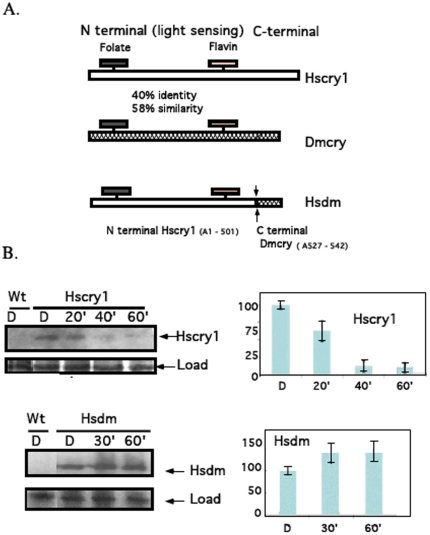
*HSCRY1* constructs and expression in transgenic *Drosophila*. Full-length *HSCRY1* cDNA was ampified by PCR and cloned into the pP(UAST) vector (Rubin et al. 1985) downstream of the UAS activator. Primers used for cloning were: N- terminal; ccc ccc gaa tcc ttt ttt atg ggg gtg aac gcc gtg cac and C - terminal; ccc ccc tct aga cta att agt gct ctg tct. For *HSCRY1− Dmcry* fusion constructs (*Hsdm*) the c-terminal domain of *HSCRY1* was replaced with a short c-terminal fragment of *Dmcry*. Primers were N-terminal; ccc ccc ctc gag atg ggg gtg aaac gcc gtg cac and C-terminal: ccc ccc tct aga cta cac cac gtc ggc cag cca gaa gaa ctg acc cac ttc ctc ctc gtt tgc cag aag acc tag tcc. The fusion junction is at A501 of HsCRY1, to the peptide 527NEEEVRQFFWLADVV* of DmCRY. At least two independent insertion lines for each construct were analyzed for all tests. A. Sequence homology of light-sensing and C-terminal domain segments of HsCRY1 and DmCRY. B. Western blot analysis of expression in flies. Expression was induced from the UAS upstream promoter element under the control of *tim-Gal4* and analyzed in F1 heterozygote expressing progeny as described [9]. Anti - HsCRY1 antibody was as described [9]. Approximately 10 flies were used per sample lane and protein concentration was quantitated by Bradford assay before loading - see [9]. Left panels represent Western blots of indicated constructs and right panel indicates quantitation – error bars are SD of three independent measurements. Flies were dark adapted for 48 hours prior to trials. Time course refers to time in white light (150 µmolm^−2^ sec^−1^, fluorescent Philips ‘cool white’ bulbs). Load represents Coomassie stain of total protein from the gels blotted to PVDF transfer membranes for the Western blots.

To determine possible biological roles of HsCRY1 in a heterologous expressing system, two distinct constructs comprising the *Hscry1* gene were cloned into a *Drosophila* expression vector. One construct comprises full-length *Hscry1* [9] and in a second construct (*Hsdm*), the C-terminal domain of *Hscry1* is replaced with that of DmCRY (Fig. 1a) to allow for possible improvement in interactions with *Drosophila* signaling partners. Both constructs were fused downstream of the UAS promoter element and introduced into flies by established methods [27]. Constructs were introduced into both wild type (wt) and *cry^b^* mutant [28] flies and expression induced from the UAS element by crossing to *tim*-Gal4 expressing flies [29]. In F1 progeny, *Hscry1* expression is therefore targeted to all of the clock cell types where timeless is expressed.

Expression of HsCRY1 was verified by Western blot analysis in dark adapted flies using anti-HsCRY1 antibody in F1 progeny lines (Fig. 1b). Upon transfer to light, HsCRY1 levels dropped rapidly, and continued to decline, such that within 40 minutes less than 20% of initial protein levels remained, consistent with previous reports [9]. This response is similar to that for DmCRY [8] and *Arabidopsis* CRY2, whose light activated form is recognized and targeted via the E3 ubiquitin ligase COP1 to the proteasome upon light activation [30]. Such light activated degradation was not observed for HsDM protein, whose levels actually increased somewhat on transfer to light (Fig. 1b), suggesting that the hybrid protein may not adopt a conformation that leads to protein instability/degradation following light activation.

### HsCRY1 does not complement the circadian phenotype of DmCRY

We next performed phenotypic analysis of transgenic flies to evaluate possible biological roles of HsCRY1 in the functioning of the fly circadian clock. Endogenous DmCRY acts as a light input to the circadian clock [28], [31]. In contrast to wild type (wt) flies, *Dmcry* mutant flies remain rhythmic in continuous high light due to loss of the light-dependent damping and of the free-running circadian rhythm that would normally occur in wild type [31], [32]. However, flies expressing either *Hsdm* or *Hscry1* in a cry^b^ genetic background maintained robust rhythmicity in LL over a period of several days (Table 1) with a period length slightly longer to that of wt flies. Therefore, HsCRY1 does not appear to complement DmCRY in the regular functioning of the *Drosophila* circadian clock. Most likely HsCRY1 does not recognize *Drosophila* clock proteins.

**Table 1:** Heterologous CRY expression does not restore a wild-type arrhythmic phenotype to *cry^b^* mutant flies.

Light conditions	Genotype	*cry* transgene present,( ): no driven expression	Total flies (n)	Rhythmic flies (%)	Period (h)			Power			Activity		
LL	*yw;;cry^b^ ss*		15	80	24,8	±	0,2	74	±	7	20	±	3
	*w 10F;;cry^b^ ss*	*(HsDmcry)*	14	93	25,5	±	0,2	115	±	9	22	±	2
	*yw/w;tim-gal4/+*		16	6	27,0			22			14		
	*yw/w;tim-gal4+/;2M cry^b^ ss/cry^b^ ss*	*HsDmcry*	14	71	25,0	±	0,5	88	±	17	26	±	4
	*w 10F/yw;tim-gal4/+;cry^b^ ss*	*HsDmcry*	19	100	25,2	±	0,2	117	±	6	27	±	2
	*w/yw;tim-gal4/26.9A;cry^b^ ss*	*Hscry1*	12	100	25,9	±	0,2	95	±	14	32	±	2
	*yw;;cry^b^ ss*		12	100	24,9	±	0,2	101	±	10	19	±	4
	*W*		11	9	25,0			65			47		
	*w;;31.2A cry^b^ ss*	*(Hscry1)*	6	100	24,8	±	0,5	86	±	9	18	±	4
	*w;tim-gal4/+;31.2A cry^b^ ss/cry^b^ ss*	*Hscry1*	16	81	25,0	±	0,2	108	±	11	40	±	6
	*w;26.9A;cry^b^ ss*	*(Hscry1)*	10	60	24,7	±	0,3	65	±	11	30	±	6
	*w;tim-gal4/26.9A;cry^b^ ss*	*Hscry1*	16	81	25,3	±	0,2	104	±	9	22	±	2
	*w 26.13C;;cry^b^ ss*	*(Hscry1)*	10	100	24,8	±	0,2	92	±	11	15	±	1
	*w 26.13C/+;tim-gal4/+;cry^b^ ss*	*Hscry1*	16	100	25,3	±	0,2	113	±	9	18	±	2
	*w;26.16;cry^b^ ss*	*(Hscry1)*	11	100	25,1	±	0,1	94	±	10	12	±	2
	*w; tim-gal4/26.16;cry^b^ ss*	*Hscry1*	15	100	25,6	±	0,1	120	±	7	17	±	1
	*w;41.4;cry^b^ ss*	*(Hscry1)*	9	67	25,0	±	0,2	99	±	9	18	±	2
	*w; tim-gal4/41.4;cry^b^ ss*	*Hscry1*	16	100	25,7	±	0,1	125	±	6	15	±	1
DD	*w;tim-gal4/+*		17	94	24,3	±	0,2	106	±	10	33	±	2
	*w;tim-gal4/+;2M*	*HsDmcry*	16	94	24,8	±	0,1	92	±	10	24	±	2
	*yw/w;tim-gal4/+;2M cry^b^ ss/cry^b^ ss*	*HsDmcry*	18	94	24,4	±	0,1	103	±	6	26	±	2
	*w 10F;tim-gal4/+*	*HsDmcry*	10	90	24,7	±	0,1	105	±	8	29	±	3
	*yw/w;tim-gal4/+;31.2A/+*	*Hscry1*	19	100	24,4	±	0,1	109	±	7	35	±	2
	*yw/w;tim-gal4/26.9A*	*Hscry1*	28	89	24,8	±	0,1	93	±	7	30	±	2

Actogramms of rhythmicity conferred by *Hscry* constructs in LL and DD. Three day old adult flies expressing HsCRY1, HsDM in both wild type and *cry^b^* mutant genetic backgrounds were entrained for 72 hours to 12 hr day/night cycles and analyzed for free running period in LL and DD for 10 days. Rhythmicity and period length are shown. The mean values of circadian period (h), associated powers (see Methods) and activities (number of events per 0.5 h) are given ± s.e.m.

### Developmental phenotype of HsCRY1 in expressing transgenic flies

**Figure 2:**
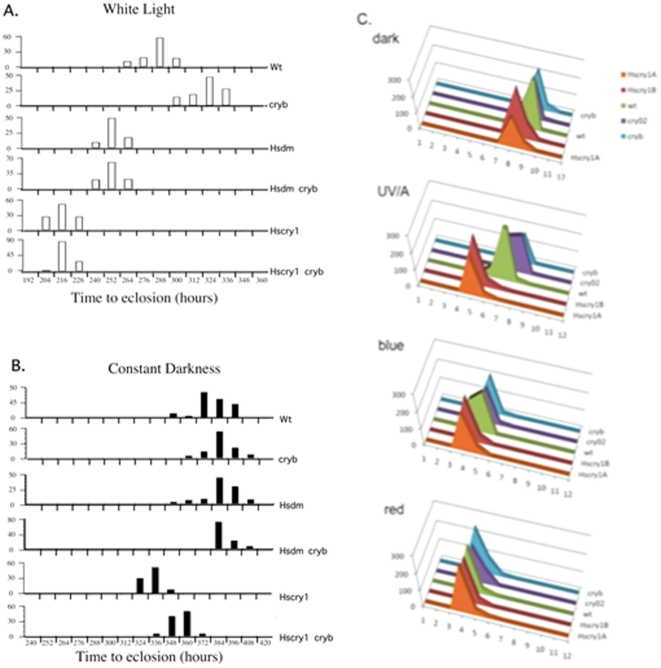
Analysis of developmental time (until eclosion) in HsCRY1 expressing transgenic *Drosophila*. For all tests, parent flies were maintained for 24 hours in a 50 ml conical falcon tube containing 10 ml of growth medium in order to lay eggs. Adult flies were then removed and tubes transferred to constant white fluorescent light (200 µmol m^−2^ sec^−1^) or continuous dark (DD) in a temperature controlled and humidity controlled plant growth chamber at 22°C. Time to eclosion was measured every 12 hours and eclosed flies counted and plotted for each time point as a percentage of total eclosed flies. Typically between 100 and 500 flies were counted per *Drosophila* line. Experiments were performed in triplicate showing qualitatively similar results (peak of eclosion); the data from a representative experiment is shown. A. Time to eclosion was plotted for LL (continuous light). Wild type controls were the non-expressing *tim-Gal4* parental lines. *cry^b^* mutant flies carried the *cry^b^* mutation in the genetic background of the *tim-Gal4* parental lines. B. Same as for ‘A’, in constant darkness (DD). C. Relative time to eclosion (x-axis) under differing wavelengths of light at 3 µmol m^−2^ sec^−1^ blue light (B), red light (R) and UV/A light (peak 380 nm) (UV). Y-axis is number of flies pooled from three independent experiments. *Hscry1*(A) flies expressed HsCRY1 under control of the *tim-Gal4* promoter, *Hscry1* (B) flies expressed HsCRY1 under the *da-Gal4* promoter; *cry^02^ i*s the null mutant allele (Dolozelova et. al. 2007).

As an alternative to clock phenotypes, a developmental phenotype known to be modified by light was investigated for a possible biological effect of HsCRY1 in transgenic *Drosophila*. It has been previously observed that the time to eclosion in developing flies is significantly reduced when eggs are hatched and maintained in constant light (LL) as compared to darkness (DD) [33]. We determined the role of both insect (DmCRY) and mammalian (HsCRY1) cryptochromes on time to eclosion in expressing flies using wt, Dmcry mutant (*cry^b^*), HsCRY1 and HsDM over-expressing transgenic flies (Fig. 2). In continuous light (LL), wt flies developed more rapidly than *cry^b^* mutant flies with the peak of eclosion occurring 1 ½ days earlier, suggesting that cryptochrome promotes this response (Fig. 2a). To test this hypothesis we compared the developmental time of both genotypes in continous darkness (DD). As reported previously, peak eclosion occurred significantly later in wt flies under DD than under LL (after 15.5 days instead of 12 days) (Fig. 2a,b). However, it occurred also later in *cry^b^* mutants (after 16 days instead of 13.5 days) (Fig. 2a,b). Therefore, DmCRY is not the primary photoreceptor implicated in this response to white light.

We next measured the effect of HsCRY1 and HsDM on eclosion time in heterozygous expressing F1 flies (Fig. 1b). In LL, developmental time was significantly shorter for both HsCRY1 (by 3 days) and HsDM (by 1.5 days) expressing flies as compared to wild type flies (Fig. 2a). Time to eclosion was also accelerated in flies that carried the constructs in the *cry^b^* genetic background, suggesting this effect is not due to interaction between heterologous and endogenous cryptochromes. Interestingly, HsCRY1 did also accelerate development in DD (Fig. 2b), although to a reduced extent as compared to LL. Wavelength dependence of the eclosion phenotypes mediated by HsCRY1 and DmCRY, respectively, was investigated in near UV (peak at 380 nm), blue (peak at 450 nm) and red (peak at 660 nm) light (Fig. 2c). Here, HsCRY1 was either expressed under control of the *timeless* (*tim*) promoter or under the control of the *daughterless* (*da*) promoter (using the *uas-Gal4* expression system). The *da* promoter is a strong promoter expressed in all cell types [34]. ensuring HsCRY1 expression throughout the fly and not just in cell types expressing the clock gene *tim*. As a further negative control, *tim-gal4*×*uas-Hscry1* flies were tested in a *cry* knockout mutant background (*cry^0^*; [32]). As in previous experiments (Fig. 2a,b), there was acceleration of time to eclosion in HsCRY1 expressing lines (peak activity is one day in advance) even in complete darkness, both in the case of *da-Gal4*×*Hscry1* and *tim-Gal4*×*Hscry1* F1 lines. This indicates a light-independent role of HsCRY1 in the acceleration of larval development in addition to a light-dependent one. Interestingly, the different HsCRY1 expressing lines grown in red light showed no significant difference in eclosion times (peak after 11 days for all lines). By contrast, responsiveness in blue light is accelerated in HsCRY1 expressing lines (peak one day in advance of wt and *cry* mutants). Surprisingly, the response in HsCRY1 expressing lines was even more pronounced at shorter wavelengths of light (near - UV) than at similar intensities of blue light, with peak of eclosion occuring two days in advance of wt and 3 days in advance of *cry^b^* and *cry^0^* mutants (Fig. 2c).

Taken together, these results indicate that the effect of HsCRY1 in this developmental response occurs to a large extent independently of light (as seen in flies kept in complete darkness). However, the enhanced response of HsCRY1 expressing flies as compared to wt or *cry* mutant flies in white, blue and UV/A, but not in red light, is consistent with an additional light activation of HsCRY1.

### Transcriptome analysis of *Hscry1* expressing transgenic flies

Mammalian Type II cryptochrome has been shown to function as a transcriptional repressor in mammalian cell cultures [21] and therefore likely to also bind to promoter elements and provide transcriptional phenotypes in heterologous systems such as *Dr*osophila. Therefore, expression profiling was performed in *Drosophila* using pupa no older than 24 hours post - 3d instar larva; this developmental stage was chosen since HsCRY1 has a profound effect on the time to eclosion (see above). Microarray analysis was performed using Affymetrix Genechip *Drosophila* 2.0 arrays (see methods). We compared *cry^0^* (*Dmcry−*/*Hscry−*), HsCRY (*Dmcry+/Hscry+*) and wild-type (*Dmcry+/Hscry1−*) flies, in the dark (D) or after a two hours dark to blue light transition (L). Among a total of over 18,500 total transcripts, genes with statistical differences in hybridization signals of two-fold or more were selected.

**Figure 3:**
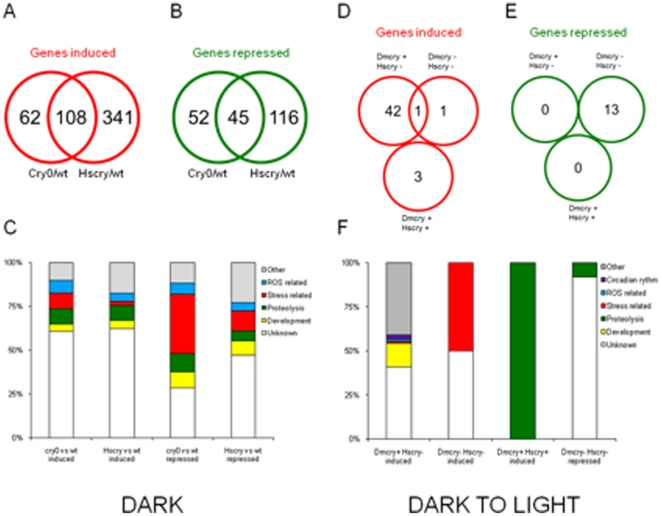
Transcriptome analysis of cryptochrome dependent gene expression in developing pupa one day after third instar larval stage. A, B Venn diagram of genes either upregulated (red) or downregulated (green) in the indicated strains under conditions of complete darkness. Comparison were performed in triplicate biological samples and analysed for statistically different expression patterns according to established programming techniques (see methods). C. Graphical representation of categories of genes either repressed or activated by cryptochromes in the indicated comparisons. All comparisons are for strains maintained in complete darkness (DD) from the time of egg laying. D,E. Venn diagrams of genes up-regulated or down-regulated in the indicated strains (wt, *cry^0^*, or *Hscry1* transgenics) after transition from dark to two hours blue light at 60 µmol m^−2^ sec^−1^. F. Graphical representation of categories of genes either repressed or activated by cryptochromes in the indicated comparisons.

In the dark, 449 genes were induced and 161 genes were repressed in HsCRY1 expressing lines in comparison to wt (D) (Fig. 3a,b). Therefore, as predicted, mammalian Type II cryptochrome has considerable effect on transcription in this heterologous organism and this function can occur independently of light. Transcriptome analysis also produced the more unexpected finding that DmCRY likewise mediates considerable transcriptional responses in the absence of light (D), in contrast to the light-dependent mechanism underlying DmCRY clock function. In the dark, 267 genes were differentially expressed in *cry0* in comparison to wt, showing the importance of DmCRY in the regulation of gene expression in complete darkness (Fig. 3a,b). Importantly, this regulation occurs by pathways independent of its role in the circadian clock under these conditions of arrhythmicity.

A comparison of genes regulated by HsCRY1 and DmCRY showed a significant proportion (154) of common genes, thereby validating this approach with respect to identifying cryptochrome regulated transcripts rather than background changes in gene expression patterns unrelated to cryptochrome function. Interestingly, genes that were regulated by both DmCRY1 and HsCRY1 in DD were often regulated in the opposite manner, for example 108 were induced and 45 were repressed in both *cry^0^* and HsCRY1 expressing flies, indicating HsCRY1 acted on the whole as inducer and DmCRY as repressor of the same genes (Fig. 3a,b). The same was true of the overall majority of regulated genes (HsCRY1 functioned primarily as inducer, DmCRY primarily as repressor in the dark) (Fig. 3a,b). When analyzing the function assigned to the selected genes using *Drosophila* databases [35], we found many involved in development and the related function of proteolysis that occurs in the course of metamorphosis (see [36] and references therein) (Fig. 3c). Our analyses also revealed an unexpectedly high proportion of genes implicated in stress response or related to ROS (reactive oxygen species), especially among genes down-regulated in both HsCRY and *cry^0^*. It therefore seems probable that HsCRY1 and DmCRY may share some common signaling pathways, and that HsCRY1 has a dominant negative effect by binding non-productively and thereby sequestering common signaling intermediates. It also seems likely that responsivity to stress may be a hitherto unrecognized role of cryptochromes that is conserved across species lines.

We next determined the possible light responsivity of cryptochrome mediated transcriptional events. A comparison of gene expression levels before and after a rapid two hour blue light exposure was undertaken (Fig. 3d,e). Comparison of expression levels showed relatively few differences in either wt flies, *cry^0^* mutants or *Hscry1* transgenics, consistent with the expectation that light-regulated gene expression is likely not a major feature during *Drosophila* development. We nevertheless obtained 43 genes that were up-regulated subsequent to light exposure in the wt (Fig. 3d). Significantly, only one of these was also induced in the *cry^0^* genetic background. Furthermore, of the 13 genes down-regulated by light in *cry^0^* mutants (Fig. 3d), 6 were up-regulated and the remainder showed no change in the wt genetic background. These results indicate that blue light induction of gene expression under these conditions in the wt was almost entirely dependent on DmCRY (Fig. 3c,d). In the case of *Hscry1* transgenics, a moderate light responsivity was observed with 3 genes induced by blue light, all of them distinct from those induced in wt and *cry^0^* genotypes. Therefore, the presence of HsCRY1 leads to light-dependent transcription in *Drosophila* by pathways independent of DmCRY mediated expression. Furthermore, the fact that DmCRY-dependent transcriptional regulation was absent in Hscry1 transgenics demonstrates that there is interference of HsCRY1 with regular DmCRY-regulated transcription. It is likely that common signaling pathways may exist with common signaling intermediates, consistent with the antagonistic roles of HsCRY1 and DmCRY transcriptional activity in the dark (Fig. 3a,b).

**Figure 4:**
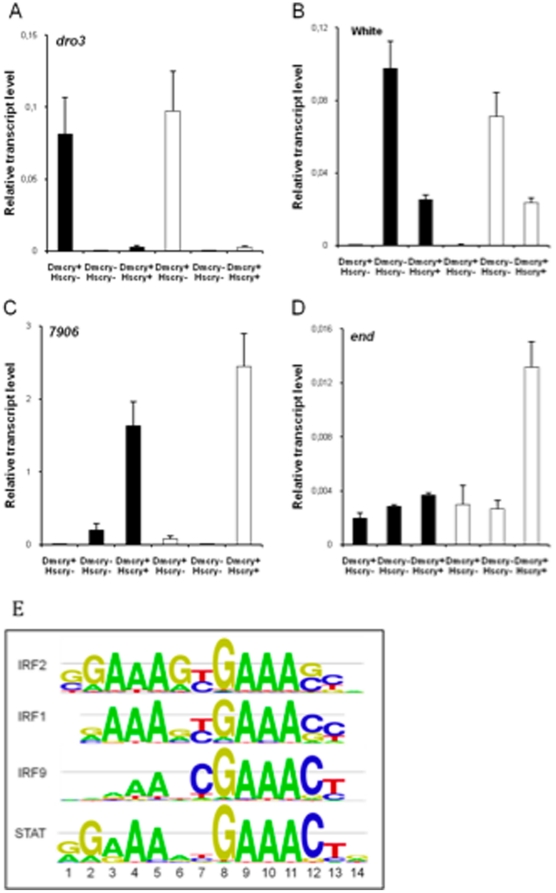
Verification of expression of indicated genes by qPCR and consensus promoter elements involved in cry regulation. Solid rectangles represent expression levels in dark, white rectangles expression after two hours of blue light. A: *dro3*, B: *white*, C. *7506*; D. *end*. E. Transcription factor binding sequence DNA motifs conserved in cryptochrome regulated promoters of *Drosophila* as obtained using *Drosophila* cisTargetX (Aerts et al., 2010), the size of the letter indicates the probability to find the corresponding base at the different positions (adenine (**A**), cytosine (**C**), guanine (**G**) and thymine (**T**)).

To verify and confirm the results obtained by microarray analysis, 4 representative cryptochrome-dependent genes were selected. *Drosomycin3* (*Dro3*, CG32283), *White*(CG2759), *Endopeptidase* (*End*, CG6467), and the gene CG7906 (7906), that encodes an unknown protein, were selected for a quantitative analysis of transcript changes (Fig. 4). As expected, in comparison to the wt, *Dro3* was down-regulated in the *cry^0^* and *Hscry* samples in the dark and in the light (Fig. 4a). Conversely, *White* transcripts were down-regulated in the wt both in the dark and in the light (Fig. 4b). As found during microarray experiments, *7906* showed a specific expression pattern with transcripts that were induced by light in the wt but down-regulated in *cry^0^* mutants in the same conditions (Fig. 4c). We also tested the expression of one of the 3 genes up-regulated by light in the HsCRY1 expressing flies. As expected, transcripts of *End* accumulated only in *Hscry1* samples after a dark to light shift (Fig. 4d). The complete list of genes differentially expressed in our microarray experiments have been deposited in the online accessible database ArrayExpress.

In terms of functions, the genes regulated by light in the wt were related to development, and to a lesser extent to circadian rhythms and to stress and ROS (Fig. 3f) as has been observed for expression in the dark (Fig. 3c). Furthermore, among the 13 genes down-regulated by light in *cry^0^* mutants, 4 belong to the Osiris [37] family which may be linked to immune responsiveness (*Osi6*, *Osi14*, *Osi19* and *Osi20*). Of these, 3 are up-regulated by light in the wt (*Osi6*, *Osi19* and *Osi20*) and none are regulated by light in the presence of HsCRY1, indicating that HsCRY1 also in some way antagonistically interacts with this response pathway. The 3 genes induced specifically in *Hscry1* transgenics in response to blue light (CG6467, CG11912, CG10475), were described as serine-type endopeptidases.

In an attempt to identify possible cis-acting promoter elements linked to cryptochrome regulation, we searched for conserved motifs known to be targets of transcription factors by the program cisTargetX [38] No representative motifs were found among the many genes differentially expressed in the dark through either DmCRY or HsCRY1 action. However, the analysis of light regulated genes revealed that among the 43 genes up-regulated by light in the wt, 15 had promoters containing a motif that could be recognized by IRF2, 12 by IRF1, 7 by IRF9 (Interferon Regulatory Factor) from mouse [39] (Fig. 4e) implicated in the mammalian antiviral immune response. In addition, 5 genes up-regulated by light contained the motif for the ‘signal transducer and activator of transcription’ (STAT) transcription factor from *Drosophila* (Fig. 4e), which are involved in *Drosophila* innate immunity against viruses. Further, consistent with these results, among the 13 genes down-regulated by light in *cry^0^* mutants, 4 (including 3 *Osiris*) possess the sequence recognized by IRF2 and 2 the sequence recognized by STAT, and all are induced in the wt under the same conditions. Since induction of gene expression occurs within two hours subsequent to the onset of illumination, and since all of these genes are regulated by DmCRY, these promoter element binding factors are likely to be novel partners on the light signaling pathway of DmCRY that have no role in the circadian clock. Significantly, almost all of these genes are repressed (prevented from being activated) by the presence of HsCRY1, which, as a consequence may also be interacting directly and somehow sequestering this conserved transcription factor. The fact that so many of the promoters induced by light contain conserved motifs further reinforces the validity of the present results in identifying targets of cryptochrome action in flies.

In summary, transcriptome analysis has revealed that Type II (mammalian HsCRY1) cryptochrome regulates transcriptional activity in *Drosophila* by (principally) light-independent, but also light-dependent mechanisms, consistent with the role of light in the eclosion phenotypes presented above. More unexpectedly, the endogenous *Drosophila* (Type I) DmCRY also regulates considerable light-independent transcriptional activity in *Drosophila*, in marked contrast to its light-dependent transcriptional role in the circadian clock. Both HsCRY1 and DmCRY are implicated in expression of many genes with no known clock function, including those related to stress responsivity, which may represent a new area of interest in cryptochrome research in this organism. Possible direct targets of cryptochromes include families of transcription factors binding to conserved promoter elements (IRF, STATs) that may be recognized by both HsCRY1 and DmCRY.

### Biochemical and photoresponsive characteristics of the isolated HsCRY1 protein

**Figure 5:**
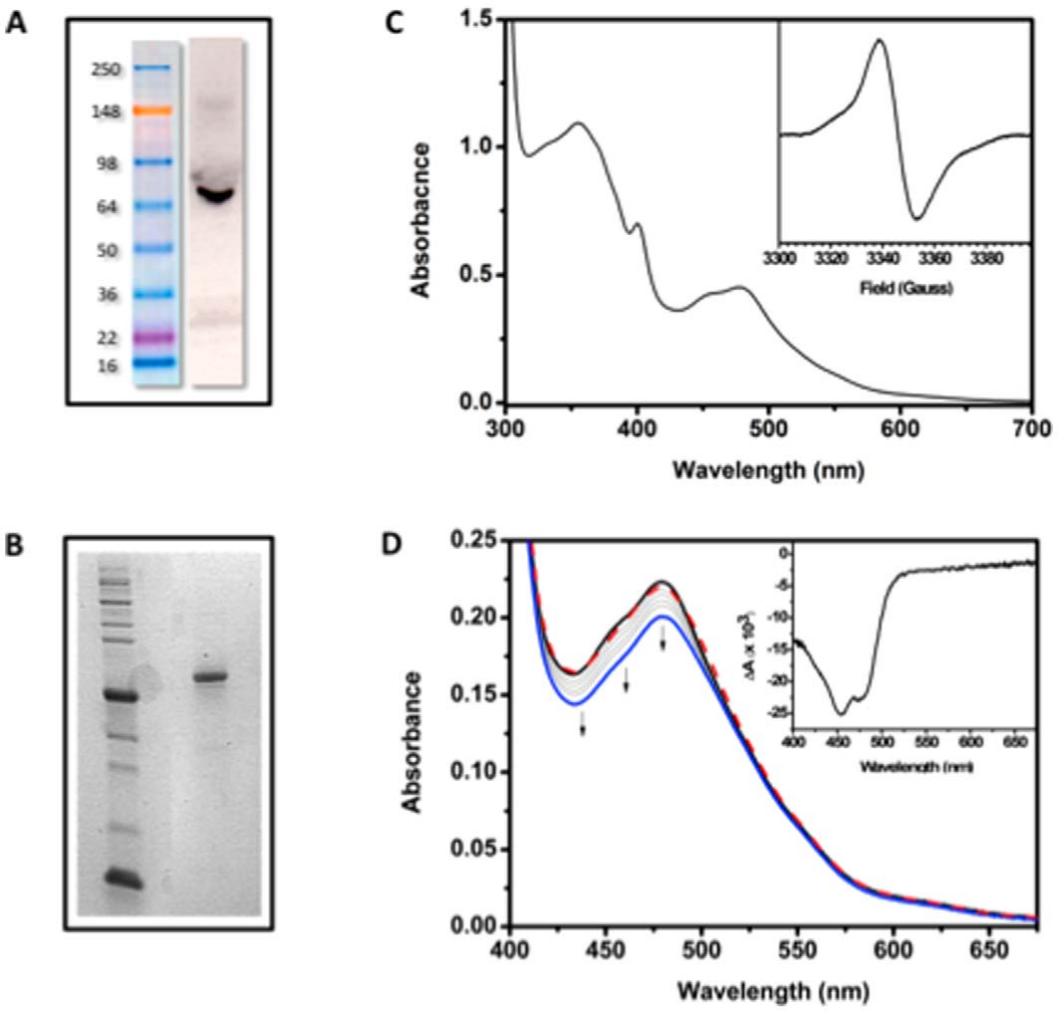
Purification and characterization of HsCRY1. A. Western blot confirming the expression of HsCRY1 using the *Homo sapiens* CRY1 polyclonal rabbit antibody. B. **Coomassie-stained** SDS PAGE of purified HsCRY1 (66 kDa). C. UV-visible spectrum of purified HsCRY1. Inset: Averaged cwEPR spectrum of a ∼290 µM sample of HsCRY1. The peak-to-peak linewidth is 15.4 G at a g-value of 2.0042, which indicates an anionic semiquinone. D. UV-visible absorbance spectra representing the photoreduction of HsCRY1 in the presence of 20 mM DTT. The sample was exposed to 100 µmol m^−2^ s^−1^ of 440–460 nm light for a maximum of 85 minutes. Black line - before irradiation; blue line - after 85 min irradiation; red, dashed line – reoxidation in dark (within ∼2 min). Inset: the difference spectrum (Light minus dark) showing peak reduction at 450 nm consistent with oxidized flavin.

To date, a number of different approaches have been adopted to isolate the human cryptochrome variants: HsCRY1 and 2 (Type II). However, heterologous expressed proteins from *E. coli* [40], human (HeLa) cells [41], and Sf21 insect cells [42] had low flavin occupancy and/or very low protein yield and were therefore not suitable for photochemical studies. In the present work, a *Pichia pastoris* expression system has been developed for producing quantities of native, flavin-bound recombinant HsCRY1 sufficient for *in vitro* photochemical studies (see methods). The intact full-length *Hscry1* gene was cloned into the pPICZ B *Pichia pastoris* expression vector without addition of an affinity tag or other heterologous protein sequences. Expression was induced by addition of methanol to yeast cultures (see methods) and verified by Western blot analysis with anti-HsCRY1 antibody to show a single, full length specific protein band (Fig. 5a). Purification was by DEAE column chromatography followed by further fractionation over a Resource Q column (see methods). Column fractions containing recombinant HsCRY1 protein at virtual homogeneity were recovered from the purification procedure, as determined by Coomassie staining on SDS polyacrylamide gels (Fig. 5b). Interestingly, upon analysis by UV/Vis spectroscopy, the isolated protein did not show the typical 450 nm peak of oxidized flavin as found in most purified preparations of currently known cryptochromes, but instead was purified as a red solution whose UV/vis absorption spectrum (Fig. 5c) more closely resembled that of the anionic (‘red’) semiquinone form of flavin [43]. The identity of anionic radical was confirmed by EPR (Fig. 5c inset).

In isolated DmCRY and other insect cryptochromes, the anionic species is formed subsequent to illumination and has been correlated with biological activity [9], [10], but reverts to the oxidized form upon return to darkness in a matter of minutes. By contrast, the anionic radical is surprisingly stable in Type II HsCRY1, remaining in this oxidation state for weeks (stored at −80°C) and with no obvious spectral change when kept on ice in the dark for several hours. Although certain isolated photolyases can also be purified in the radical form, this is generally as the neutral radical. Since formation of the radical species has been correlated with biological activity [9], [12] the apparent stability of this redox form can now provide a possible explanation for the ‘dark active’ role of mammalian type II cryptochromes, which would not need to be reduced by light in order to accumulate the radical form.

The isolated purified protein also had a significant peak in the near UV spectral region which could in part be due to flavin (peak at 360 nm) but also may represent some contribution from an antennae (likely folate) cofactor found in most photolyases and some cryptochromes [1], [2]. This antenna pigment contributes significant absorption in the near - UV spectral region (peak at 380 nm) in photolyases and its presence in Type II cryptochromes is consistent with reported UV sensitivity of Type II cryptochromes from butterfly during magnetoreception [44]. To determine the chromophore composition of the protein, the HsCRY1 protein sample was denatured by heating to 95°C and a spectrum taken of the supernatant (Figure S1A). Free flavin appears to have been liberated, showing the typical absorption peaks at 450 nm and at 360 nm. This allowed calculation of the extinction coefficients across the spectral range of the bound anionic semiquinone, based on *ε* = 11.3 mM^−1^ cm^−1^ for liberated, oxidized FAD at 450 nm. The sample in Fig. 5c, therefore, has a radical concentration of ∼150 µM (*ε* = 2.66 mM^−1^ cm^−1^ at 478 nm). In addition, the strong peak at around 360 nm had an intensity relative to the 450 nm peak suggesting a possible contribution from 5,10-methenyltetrahydrofolate (MTHF) as well as from FAD (Fig. 5c). This possibility was further explored by fluorescence spectroscopic techniques. As determined by the emission spectrum (excitation at 365 nm), a significant broad peak around 440–460 nm is consistent with the presence of bound folate (Fig. S1B).

One of the characteristics of cryptochromes correlated with light sensing is the ability to undergo redox reactions (photoreduction) in response to light. This requires that flavin undergo reduction from a more oxidized form (generally fully oxidized flavin) to a more reduced form (the radical form of flavin is usual for cryptochromes). Such reactions have been detected *in vivo*; in insect cell cultures expressing high levels of HsCRY1 proteins, photoreduction has been observed by both fluorescence and EPR spectroscopic techniques [9] indicative of oxidized flavin bound to HsCRY1 *in vivo*. It was therefore to be expected that at least some proportion of the purified protein *in vitro* should be bound to flavin in the oxidized form, as a condition for possible photoreduction to the (apparently active) radical form. Accordingly, to detect the possible presence of oxidized flavin in the sample, the fluorescence excitation spectrum was recorded, monitoring emission at 525 nm (Figure S1C). The resulting spectrum is somewhat broad, which is likely to a result of a combination of factors: the contribution of multiple fluorescing species including that of folate (MTHF); the relatively low extinction coefficient of protein bound flavin in cryptochromes; the relatively low proportion of oxidized flavin bound to HsCRY1. However, there is a clear shoulder at 450 nm, which is consistent with the presence of oxidized FAD in a sample otherwise dominated by the red semiquinone [10], [12], [13], [40].

We next determined whether the purified sample could undergo redox reactions (photoreduction) in response to light. Generally, such reactions are performed *in vitro* with isolated proteins in the presence of reducing agents such as DTT or 2 -mercaptoethanol [12], [13]. An anaerobic sample of HsCRY1 was passed down a 10 DG size-exclusion column (equilibrated with buffer containing 20 mM DTT) to remove any unbound FAD. Spectra where then acquired in the dark and after exposure to 100 µmol m^−2^ s^−1^ blue-light of wavelength 440–460 nm (generated by a cold halogen lamp and a 450 nm bandpass filter). Fig. 5d clearly illustrates that there is a small but significant photoreduction of HsCRY1 under these conditions, with a reduction in absorbance at wavelengths centred around 450 nm (see inset which represents the difference spectrum - dark minus light). The light induced reaction occurred over the course of 85 mins, and presumably represents the reduction of the small amount oxidized flavin bound to the fully reduced form via the anionic semiquinone. The sample was then exposed to air, drawn through a pipette a few times and consequently reoxidized in the dark (within the time taken to aerate the sample and take the spectrum). Interestingly, even at this relatively high degree of illumination, there was almost no reduction in radical flavin, which represents the majority of the bound FAD, to form the fully reduced (FADH-) form typical of photolyases. In this respect, HsCRY1 behaves like plant and *Drosophila* cryptochromes (which oscillate preferentially between oxidized and radical forms and can not be easily fully reduced *in vitro*) and unlike photolyases or the photolyase-like Cry-DASH cryptochromes [1].

In summary, HsCRY1 adopts a mixture of flavin redox states upon purification, which contain a large proportion of the anionic radical species that has been correlated with biological activity in numerous studies. Therefore, if this were indeed the signaling state, there would be no need for light activation to reach a biologically active conformation of Type II cryptochromes and could explain the light-independent role of this receptor. In addition, there is a smaller proportion of receptor with flavin bound in the oxidized form that can undergo light activation to form the radical state. This may explain the additional light resposivity of HsCRY1 in transgenic *Drosophila*. Finally, there is evidence of an additional bound cofactor, folate, which could contribute to UV sensing.

## Discussion

In the current work, we have taken a transgenic approach to determine the sensitivity to light of a Type II cryptochrome using HsCRY1 expression in *Drosophila*. A combination of developmental studies and expression profiling analysis, has shown that HsCRY1 has principally light-independent roles in flies, of which a small subset are enhanced in the light. By contrast, Type I (*Drosophila*) cryptochromes which show numerous light responsive phenotypes as extensively documented for the circadian clock [1] are shown to have significant light-independent signaling roles in addition to those that are responsive to light. These observations suggest an absence of fundamental mechanistic differences between Type I and Type II cryptochromes, and that their different responsivity to light is due to differences in the stability of the radical flavin in the protein which represents the signaling state. It is also intriguing that certain molecular targets of DmCRY may also be recognized by HsCRY1, suggesting that some conservation of cryptochrome signaling pathways may have occurred between such highly disparate organisms as flies and man.

### Acceleration of *Drosophila* Development by HsCRY1

We here describe a novel phenotype mediated both by DmCRY and by HsCRY1 in transgenic expressing *Drosophila* that results in aceleration of larval development. It should be noted that these similar phenotypes are most likely caused by distinct mechanisms (see transcriptome analysis) impacting on different aspects of larval development and should not be construed as direct physiological complementation of DmCRY mutation by HsCRY1. For the purposes of this study, the biological effects of HsCRY1 are interpreted solely as a ‘readout’ phenotype enabling assessment of light dependent and/or independent biological function of the photoreceptor within a living organism.

The acceleration of eclosion (developmental timing) that is mediated by HsCRY1 occurs in the absence of light (complete darkness) consistent with light-independent activity of HsCRY1 in transgenic *Drosophila* as in mammals. However, the effectiveness of HsCRY1 in mediating this response is enhanced in white light (Fig. 2a) indicating an additional level of light sensitivity of this reaction. Wavelength dependence of the eclosion phenotype shows that in HsCRY1 expressing flies, acceleration of development as compared to wt controls is observed in blue light and is even more pronounced in UV/A (380 nm) as compared to DD. However, in red light, although developmental timing is accelerated in comparison to DD, there is no effect of cryptochrome, either of DmCRY or HsCRY1, on this phenotype. Therefore, sensitivity to light conferred by Type II cryptochromes corresponds to the absorption spectrum of the bound flavin chromophore and is confined to the blue/UV-A spectral region, consistent with the wavelength sensitivity of type I (*Drosophila*) cryptochrome [8], [9].

### Transcriptome analysis

Transcriptome analysis has supported these developmental observations by providing evidence of both light-dependent and light-independent transcriptional roles of HsCRY1 in developing pupa. The fact that many of the same genes are regulated by both HsCRY1 and DmCRY but in opposing manner (Fig. 3) contributes to the validation of this approach as a test for cryptochrome-dependent function, since the same genes are regulated differently dependent on the cryptochrome context. This also suggests that heterologous HsCRY1 is implicated in some of the same response pathways as DmCRY, possibly by targeting some of the same transcription factors non-productively and thereby sequestering them. In the case of light-dependent transcriptional responses, these are obtained within two hours of blue light illumination and may well result from direct interaction of cryptochromes with transcription factor binding to the regulated promoters. In this respect, identification of recurring motifs in genes regulated by both HsCRY1 and DmCRY (bound by an IRF-like transcription factors in mice and STAT transcription factors in flies) further supports the validity of the approach and suggests possible targets for both HsCRY1 and DmCRY that may have homologs in both flies and mammalian systems.

Transcriptome analysis also produced the more unexpected finding that DmCRY can mediate considerable transcriptional responses in the complete absence of light, in contrast to the light-dependent mechanism underlying DmCRY clock function. Although a few isolated prior reports of a light-independent role for cryptochrome in the fly circadian clock exist [10], [14], [45], these roles involve uniquely clock entrainment in certain peripheral tissues or to temperature cycles. In the present work, by contrast, significant transcriptional activity mediated by DmCRY occurs even in the dark, reminiscent of findings for *Arabidopsis* cryptochrome, which also showed significant light-independent transcriptional responsivity [46]. Both Type I (*Drosophila*) and plant cryptochrome-1 show robust light-responsivity and undetectable constitutive activity for a number of responses in the absence of light. The observed light-independent response of DmCRY may therefore be indicative of a small pool of ‘activated’ cryptochrome that accumulates in the dark, possibly involving the radical flavin state in equilibrium with the inactive (oxidized) flavin state (see arguments developed below). Alternatively, there may be more than one means of cryptochrome function in signaling pathways, one of which can occur in the absence of light.

A further unexpected finding from transcriptome analysis is the observation that both HsCRY1 and DmCRY regulate many genes that have been associated previously with abiotic and biotic stress, disease resistance, and responsiveness to oxidative stress [35]. In addition to several Osiris genes that seem to be regulated by cryptochrome (DmCRY and HsCRY1), which may involve immune responsiveness, a link to biotic stress has been proposed involving the Toll-like receptor pathway and the expression of *Drosomycin* (*Dro*) genes [37], consistent with our observation that *Dro3* is differentially regulated by DmCRY (Fig. 4a). This activity is apparently unrelated to a role in the circadian clock, and may therefore be among the emerging novel functions of DmCRY that have recently been reported [25]. A role for cryptochrome in stress response has not yet been described in *Drosophila*, however, in mammals recent studies have raised a potential role for CRY in apoptosis and in the response to stress [47], [48]. In plants, the role of CRY in programmed cell death (PCD) and in the response to various abiotic and biotic stresses has also been described in several studies [49]–[52]. Therefore, the present work opens up the prospect of a new field of *Drosophila* cryptochrome function and its role in stress. Given that similar pathways are regulated by HsCRY1 in this transgenic system, these may furthermore involve conserved pathways and possible homologous molecular targets in mammals and other systems.

### Implications for animal cryptochrome signaling mechanism

In previously proposed mechanisms for light activation of both plant and animal cryptochromes has involved photoreduction from oxidized flavin to form a relatively stable radical intermediate, which is the presumed signaling state [9], [12], [13]. Conformational change related to signaling has been shown to require radical formation in *Arabidopsis* cryptochrome [53] and a form of light-induced conformational change requiring the presence of the radical form is proposed as the basis for signaling also in insect cryptochrome [54]. In all cryptochromes and photolyases investigated to date, flavin photoreduction occurs by electron transfer to excited state flavin from a nearby trp residue in the protein, which in turn is reduced through a chain of at least three conserved trp residues (known as the trp triad) by external reductant at the protein surface (see [1] and references therein). Return to darkness restores oxidized flavin, which resets the system to the default ‘off’ signaling state, which can be reactivated by subsequent further illumination [55]. Recently, this notion has been challenged by the observation that mutations in conserved trp residues of this electron transfer chain that prevent photoreduction of insect cryptochromes *in vitro* still show biological activity *in vivo* [25], [54]. However, this confusion is resolved by the observation that photoreduction *in vivo* still occurs in such mutants, most likely by alternative electron transfer pathways within the protein or in the extracellular environment (see discussion in [9]).

In the present work, the observed stability of the anionic radical flavin in the isolated purified HsCRY1 photoreceptor is entirely consistent with a signaling mechaism requiring flavin radical formation for cryptochrome activation. Given this property *in vitro*, a significant proportion of Type II cryptochrome would occur in the anionic flavin state in the reducing intracellular environment *in vivo*, as has in fact been observed in prior studies [9]. The accumulation of anionic radical activated ‘signaling on’ state would explain the constitutive function of mammalian Type II cryptochromes in the absence of light, whereas the remaining proportion of oxidized flavin form that can be photoreduced by light would explain additional light-responsivity. By contrast, both DmCRY and plant cryptochromes occur in a largely oxidized flavin state *in vivo* [9], [12], [56] and require photoreduction by light to produce the radical state proposed for signaling. Interestingly, an apparent Type II cryptochrome from the garden warbler [57], was reportedly isolated in the oxidized form and only transiently accumulated radical in the course of photoreduction, suggesting greater light responsivity than in their mammalian counterparts. In the latter cases, a small proportion of radical that may accumulate intracellularly in the absence of light could explain any residual minor light-independent response characteristics of these and other types of cryptochromes.

In summary, we demonstrate that mammalian (Type II) cryptochrome is capable of mediating light-independent phenotypes in transgenic expressing *Drosophila* related to gene expression and timing of development, some of which can be further stimulated by light. We furthermore provide evidence that DmCRY can mediate novel transcriptional responses in flies in the absence of light, in contrast to the light-dependent mechanism underlying DmCRY clock function. Therefore, the conceptual distinction between light sensing and light-independent cryptochromes may not imply fundamental differences in mechanism but instead be a reflection of the different equilibrium states reached between activated (anionic radical) and inactive (oxidized) resting states of the receptor that can be further modified by light. In mammals, cryptochromes are expressed in most tissue types and also occur in peripheral locations that are penetrated by light. Therefore, future studies of HsCRY1 signaling pathways may lead to identification of novel light-dependent cryptochrome responses in man that have yet to be discovered.

## Materials and Methods

### Constructions and preparation of transgenic flies

Full- length *Hscry1* cDNA was ampified by PCR and cloned into the pP(UAST) vector [27] downstream of the UAS activator. Primers used for cloning were: N- terminal; ccc ccc gaa tcc ttt ttt atg ggg gtg aac gcc gtg cac and C - terminal; ccc ccc tct aga cta att agt gct ctg tct. For HsCRY1 - *Dmcry* fusion constructs (*Hsdm*) the c-terminal domain of *Hscry1* was replaced with a short c-terminal fragment of *Dmcry*. Primers were N-terminal; ccc ccc ctc gag atg ggg gtg aaac gcc gtg cac and C-terminal: ccc ccc tct aga cta cac cac gtc ggc cag cca gaa gaa ctg acc cac ttc ctc ctc gtt tgc cag aag acc ta g tcc. The fusion junction is at A501 of HsCRY1, to the peptide 527NEEEVRQFFWLADVV* of DmCRY. At least two independent insertion lines for each construct were analyzed for all tests. HsCRY1 antibodies were as described [9].

### Circadian actogramm experiments

Actogramms of rhythmicity conferred by *Hscry* transgenic flies in LL and DD. Three day old adult flies expressing *Hscry1*, *Hsdm* in both wt and *cry^b^* mutant genetic backgrounds were entrained for 72 hours to 12 hr day/night cycles and analyzed for free running period in LL and DD for 10 days. Rhythmicity and period length are shown.The mean values of circadian period (h), associated powers, and activities (number of events per 0.5 h) are given ± s.e.m.

### Tests for eclosion

For all tests, parent flies were maintained for 24 hours in a 50 ml conical falcon tube containing 10 ml of growth medium in order to lay eggs. Adult flies were then removed and tubes transferred to constant white fluorescent light (200 µmolm^−2^sec^−1^) or continuous dark (DD) in a temperature controlled and humidity controlled plant growth chamber, 22°C. Time to eclosion was measured every 12 hours and eclosed flies counted and plotted for each time point as a percentage of total eclosed flies. Typically between 100 and 500 flies were counted per *Drosophila* line. A. Time to eclosion was plotted for LL (continuous light). Wild type flies were the non-expressing *tim-Gal4* parental lines. *cry^b^* mutant flies were flies in which the *cry^b^* mutation was introduced into the *tim-Gal4* parental lines. The genetic backgrounds of wild type and *cry^b^* mutant flies are therefore comparable.

### RNA Isolation and RT-PCR Analysis

Total RNA was prepared, using the Trizol reagent (Invitrogen, Carlsbad, CA, USA), following the manufacturer's instructions. RNA (1 µg) was treated with RQ1 RNase-Free DNase (Promega, Madison, WI, USA) and reverse-transcribed using random hexamers and SUPERSCRIPT™ III First-strand kit (Invitrogen, Carlsbad, CA, USA) following the manufacturer's recommendations. The expression levels of *Dro3* (CG32283) and *WHITE* (CG2759), *7906* (CG7906) and *End* (CG6467), were determined using the corresponding primers: *dro3-5*: GCACACTGTTTTGGCACG, *dro3-3*: GGCGGCACTTTTCTCC; *white-5*: AGGTGCTGAAGAAGCTGTC, *white-3*: ACCCACGTAGGAAAAGAAGTC; *7906-5*: TGTGACCAAGGTGATGCAG, *7906-3*: GTGTTGTTTCGGATGGTTCAG; *end-5*: CATCTCCCTGATCAAGATCCC, *end-3*: AGATTGGTAGCCACACCAC. Relative mRNA abundance was calculated by using the comparative cycle threshold (ΔCt) method and normalized to the corresponding *RP49* (CG7939) gene levels using the following primers: RP49-5: CGTTTACTGCGGCGAGAT; *RP49-3*: CCGTTGGGGTTGGTGAG. Equivalent efficiencies between the different probes and our internal standard were observed during the PCRs.

### Microarray analysis

Total RNA was prepared, using the Trizol reagent (Invitrogen, Carlsbad, CA, USA), following the manufacturer's instructions. 500 ng of total RNA from each samples were processed, labeled and hybridized to Affymetrix Genechip Drosophila 2.0 arrays according to the manufacture's suggestions (3′ IVT Express Kit User Manual, Affymetrix, Santa Clara, CA). Three Biological replicates were run for each condition. Gene-level expression values were derived from the CEL file probe-level hybridization intensities using the model-based Robust Multichip Average algorithm (RMA) [58]. RMA performs normalization, background correction and data summarization. An analysis is performed using the LPE test [59]. and a p-value threshold of p<0.05 is used as the criterion for expression. The estimated false discovery rate (FDR) of this analyse was calculated using the Bonferroni approach [60] in order to correct for multiple comparisons. All data is MIAME compliant and the raw data has been deposited in ArrayExpress, UK database (accession number pending, will be received prior to publication).

### HsCRY1 Protein Purification

The gene encoding HsCRY1 was synthesized by GenScript and codon-optimised for expression in *Pichia pastoris*, hence removal of the EcoR1 in the native *HsCRY1* cDNA. It was recloned into the EcoR1 and Xbal sites of the pPICZ B *Pichia pastoris* expression vector. This was then transformed into the SMD1168H *Pichia* host strain. Expression trials were analysed by SDS PAGE and immunodetection using the HsCRY1 polyclonal rabbit antibody (Thermo Scientific, Figure 1a). The recombinant protein was expressed by growing 4 L of culture in BMMY media at 30°C and by methanol induction (0.5% v/v) every 24 hours. The cells were harvested after 48 hours by centrifugation, resuspended in 50 mM potassium phosphate pH 7.5, 20% glycerol, 0.05% Triton X-100, 40 µM FAD, 1 mM PMSF, and lysed by passing twice through a cell disruptor (TS-series, 1.1 kW, Constant Systems Ltd) at 15,000 psi. The supernatant was clarified by centrifugation and loaded on a DEAE-sepharose column equilibrated with Buffer A: 50 mM potassium phosphate pH 7.5, 10% glycerol and 0.05% Triton X-100. The column was eluted with a linear gradient of Buffer A containing KCl from 0 to 300 mM. Fractions containing Hscry1 where pooled, dialysed against Buffer A overnight, and loaded onto a 6 ml Resource-Q column. This column was eluted against a linear gradient of Buffer A containing 0 to 500 mM KCl, and the fractions of purified HsCRY1 (SDS PAGE, Figure 1b) were dialysed once more against buffer A to remove the salt. The sample was concentrated by ultrafiltration through a 30,000 MWCO PES membrane.

### EPR Analysis

Solutions of HsCRY1 (250 µl, ∼290 µM) in 4 mm o.d. suprasil quartz tubes (Wilmad, USA) were frozen to 77 K in liquid nitrogen. The tubes were then transferred to a Bruker E500/E580 X-band EPR spectrometer equipped with a Super High Q (SHQ) resonator wherein sample temperature was maintained at 80 K using an Oxford Instruments ESR900 cryostat coupled to an ITC503 Intelligent Temperature Controller from the same manufacturer. Accurate g-values were determined using a Bruker NMR Gaussmeter. Spectra were obtained in continuous wave mode with a microwave power of 10 µW, a field modulation frequency of 100 kHz and a modulation amplitude of 1 G.

## Supporting Information

Figure S1 A - The UV-vis absorbance spectrum of purified HsCRY1 is shown in the solid line. The dashed line, which shows the absorbance spectrum of supernatant after thermally denaturing HsCRY1 by heating to 95°C for 10 mins and centrifugation, was used to calculate the extinction coefficient. B - Fluorescence emission spectrum of purified HsCRY1 upon excitation at 365 nm. The broad peak between 440–460 nm is consistent with the presence of folate cofactor. C - Fluorescence excitation spectra of HsCRY1 monitored at an emission wavelength of 525 nm. The shoulder at 450 nm is consistent with the presence of a small quantity of oxidized flavin.
(TIFF)
