# Supplementary figures and images for: Human Cryptochrome-1 Confers Light Independent Biological Activity in Transgenic Drosophila Correlated with Flavin Radical Stability
Source: PLoS One. 2012 Mar 12;7(3):e31867. doi: 10.1371/journal.pone.0031867 (PMC3299647; doi:10.1371/journal.pone.0031867)

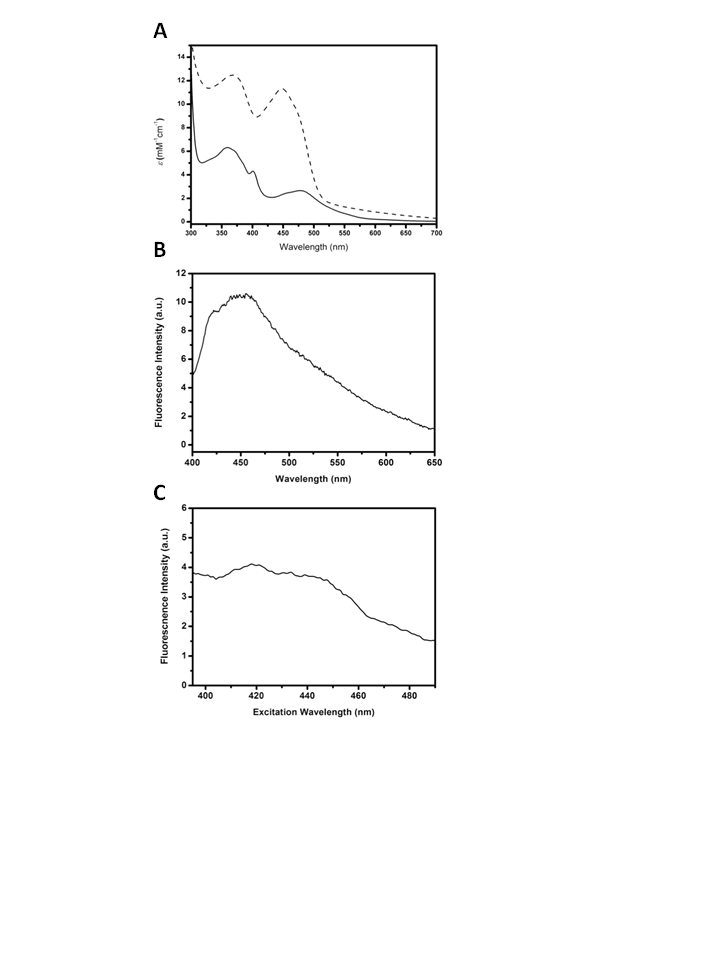

Supplement: Figure S1 — A - The UV-vis absorbance spectrum of purified HsCRY1 is shown in the solid line. The dashed line, which shows the absorbance spectrum of supernatant after thermally denaturing HsCRY1 by heating to 95°C for 10 mins and centrifugation, was used to calculate the extinction coefficient. B - Fluorescence emission spectrum of purified HsCRY1 upon excitation at 365 nm. The broad peak between 440–460 nm is consistent with the presence of folate cofactor. C - Fluorescence excitation spectra of HsCRY1 monitored at an emission wavelength of 525 nm. The shoulder at 450 nm is consistent with the presence of a small quantity of oxidized flavin. (TIFF) [file pone.0031867.s001.tif]
